# Postoperative orthostatic intolerance following fast-track unicompartmental knee arthroplasty: incidence and hemodynamics—a prospective observational cohort study

**DOI:** 10.1186/s13018-024-04639-6

**Published:** 2024-04-01

**Authors:** Ana-Marija Hristovska, Louise B. Andersen, Bodil Uldall-Hansen, Henrik Kehlet, Anders Troelsen, Kirill Gromov, Nicolai B. Foss

**Affiliations:** 1 Department of Anesthesiology and Intensive Care, Copenhagen University Hospital Hvidovre, Kettegård Alle 30, 2650 Hvidovre, Copenhagen, Denmark; 2 Section of Surgical Pathophysiology, Copenhagen University Hospital Rigshospitalet, Copenhagen, Denmark; 3 Department of Orthopedic Surgery, Copenhagen University Hospital Hvidovre, Copenhagen, Denmark

**Keywords:** Orthostatic intolerance, Unicompartmental knee arthroplasty, Postoperative recovery

## Abstract

**Background:**

Early postoperative mobilization is essential for early functional recovery but can be inhibited by postoperative orthostatic intolerance (OI). Postoperative OI is common after major surgery, such as total knee arthroplasty (TKA). However, limited data are available after less extensive surgery, such as unicompartmental knee arthroplasty (UKA). We, therefore, investigated the incidence of OI as well as cardiovascular and tissue oxygenation responses during early mobilization after UKA.

**Methods:**

This prospective single-centre observational study included 32 patients undergoing primary UKA. Incidence of OI and cardiovascular and tissue oxygenation responses during mobilization were evaluated preoperatively, at 6 and 24 h after surgery. Perioperative fluid balance, bleeding, surgery duration, postoperative hemoglobin, pain during mobilization and opioid usage were recorded.

**Results:**

During mobilization at 6 h after surgery, 4 (14%, 95%CI 4–33%) patients experienced OI; however, no patients terminated the mobilization procedure prematurely. Dizziness and feeling of heat were the most common symptoms. OI was associated with attenuated systolic and mean arterial blood pressure responses in the sitting position (all *p* < 0.05). At 24 h after surgery, 24 (75%) patients had already been discharged, including three of the four patients with early OI. Only five patients were available for measurements, two of whom experienced OI; one terminated the mobilization procedure due to intolerable symptoms. We observed no statistically significant differences in perioperative fluid balance, bleeding, surgery duration, postoperative hemoglobin, pain, or opioid usage between orthostatic intolerant and tolerant patients.

**Conclusions:**

The incidence of orthostatic intolerance after fast-track unicompartmental knee arthroplasty is low (~ 15%) and is associated with decreased orthostatic pressure responses. Compared to the previously described orthostatic intolerance incidence of ~ 40% following total knee arthroplasty, early orthostatic intolerance is uncommon after unicompartmental knee arthroplasty, suggesting a procedure-specific component.

*Trial registration*: Prospectively registered at ClinicalTrials.gov; registration number: NCT04195360, registration date: 13.12.2019.

**Supplementary Information:**

The online version contains supplementary material available at 10.1186/s13018-024-04639-6.

## Background

Enhanced Recovery After Surgery (ERAS) is a standardized, multidisciplinary, patient-centred strategy developed to address the pathophysiological challenges inflicted by surgery and anaesthesia [1]. The ERAS approach entails early postoperative mobilization, which, however, can be inhibited or delayed by postoperative orthostatic intolerance (OI), thereby increasing the risk of postoperative complications, and prolonging the in-hospital length of stay. OI is clinically defined as symptoms of dizziness, nausea, blurred vision, vomiting, visual disturbances, feeling of heat or pre-syncope/syncope [2, 3].

Postoperative OI is a complex condition with multifactorial pathogenesis that might include surgical stress response, residual anaesthesia effects, postoperative autonomic nervous system dysfunction, hypovolemia, pain and opioid use [4]. The incidence of early OI is reported to be 40–60% across several major procedures [5, 6], including total hip [7] and total knee arthroplasty (TKA) [8]. On the contrary, minor surgical procedures such as superficial breast cancer surgery, have minimal OI sequelae [9], suggesting that the severity of the inflammatory response to surgery might play an important role in the development of OI.

Globally, knee arthroplasty is one of the most performed orthopaedic procedures with a good cost-effectiveness analysis. Both TKA and unicompartmental knee arthroplasty (UKA) are currently used to treat isolated unicondylar end-stage osteoarthritis. Historically, the utilization of UKA has been restricted to ~ 10% of patients [10], but current trends are towards increasing utilization and improved outcomes [11]. UKA is a less extensive surgical procedure when compared to TKA. Furthermore, its benefits include less bleeding, postoperative pain and opioid usage, shorter in-hospital length of stay, fewer postoperative complications and reduced cost [12–16]. However, minimal data exist on OI following UKA [17].

We hypothesized that the incidence of orthostatic intolerance after UKA is lower when compared to TKA. Consequently, the aim of the present study was to investigate in detail the incidence of OI during early mobilization in a fast-track UKA setting. Secondly, we wanted to describe changes in cardiovascular function and tissue oxygenation during well-defined early mobilization.

## Methods

We conducted a prospective observational single-center study in a high-volume orthopedic surgery department. Thirty-two patients undergoing medial UKA were enrolled in the study in the period December 2019–November 2021, and 28 were included in the final analysis. Inclusion criteria were age > 18, ability to speak and understand Danish, informed and written oral consent. Exclusion criteria were known orthostatic intolerance or hypotension, cognitive dysfunction, alcohol or substance abuse or habitual use of anxiolytic, antidepressant, or antipsychotic drugs.

### Anesthesia, surgery and pain management

All patients received oral acetaminophen 1 g and celecoxib 400 mg at the ward preoperatively. Patients received spinal anaesthesia (10 mg hyperbaric bupivacaine at L2-L4) and propofol sedation at the discretion of the attending anesthesiologist. Tranexamic acid 1 g, dicloxacillin 2 g and methylprednisolone 125 mg were administered IV. Cementless mobile-bearing UKA component inserted using microplasty instruments were applied in all cases. Forced-air warming device (Bair-Hugger®; Augustine Medical, Minneapolis, USA) was used to maintain normothermia. To cover basal and surgical losses, a fixed volume regimen of 12 mL kg^−1^ isotonic Ringer acetate was administered during the first hour of surgery, followed by 6 mL kg^−1^ h^−1^ until the end of surgery. A tourniquet thigh pressure of 250 mmHg was used during the entire surgical procedure. High-volume infiltration analgesia with 200 mL 2 mg/mL Ropivacaine with 1 mg epinephrine was administered at the end of surgery[18].

### Postoperative care

Patients could drink freely in the post-anaesthesia care unit (PACU) and in the surgical ward. Postoperative pain scores were graded on a verbal rating scale VRS (0–10). If they exceeded 3 at rest or 5 during mobilization, patients received supplemental sufentanil 5 mcg at PACU and peroral morphine or oxycodone 10 mg at the ward. Postoperative pain treatment at the surgical ward included oral acetaminophen 1 g 6 h^−1^ and celecoxib 200 mg 12 h^−1^. Tranexamic acid 1 gr was repeated at 3 h after surgery. Postoperative nausea and vomiting were treated with ondansetron 4 mg PO up to 3 times day^−1^. Muscle spasms, resulting in pain and discomfort, were treated with chlorzoxazone 250 mg PO up to 6 times day^−1^.

### Orthostatic challenge

A standardized mobilization procedure was performed ~ 1 h preoperatively and repeated at 6 and 24 h after surgery, defined as the time from wound closure. The mobilization procedure included the following: patient supine rest (5 min), followed by 30° passive leg raise (PLR) (3 min) [19, 20], supine rest (5 min), sitting on the edge of the bed with feet resting on the floor (3 min) followed by standing using a walker while the patient was encouraged verbally to stand on toes and shift body weight from one leg to the other (3 min) and finally rest in a supine position (5 min). The procedure was terminated prematurely in any position if patients experienced unbearable symptoms of OI or upon a decrease of systolic arterial pressure (SAP) > 30 mmHg.

During the mobilization procedure, continuous arterial blood pressure was measured non-invasively by finger cuffs applied on the second and third finger at heart level using LiDCO Rapid (LiDCO, London, UK). The PulseCO™ method is based on principles of conservation of mass and power (pulse power analysis) and transforms the arterial waveform from pressure to a volume equivalent through a compliance and aortic volume correction maneuver. Autocorrelation of the volume waveform derives heart rate (HR) and input pulsatile volume change i.e. stroke volume (SV). Cardiac output (CO) is derived by multiplying SV by HR. Systemic vascular resistance (SVR) was calculated as ratio of mean arterial pressure (MAP) to CO. Patients with a rise in SV > 10% during PLR maneuver were defined to be preload dependent [21]. Muscle and cerebral oxygenation were recorded at 2-s intervals using Masimo Root® near-infrared spectroscopy (NIRS) with optodes placed on the biceps brachii muscle and the forehead. The Perfusion Index (PI) was measured using Masimo Root® Radical 7 pulse oximetry. Bair Hugger was not used during the hemodynamic measurements. Pain was graded using a VRS (0–10) for each body position, and patients were enquired about OI symptoms using a standardized questionnaire. Postoperative consumption of rescue opioids was registered both 6 h prior to each mobilization procedure, as well as cumulated from wound closure to each mobilization procedure to consider both the opioids' duration of action and cumulated effect. Opioid usage was calculated as opioid equivalents for both peroral and intravenous administration using an online opioid-conversion calculator (pro.medicin.dk). Remaining motor blockade was ruled out using the modified Bromage scale [22].

### Orthostatic classification

During the mobilization procedure, patients were classified as having orthostatic hypotension (OH) if they presented with a decrease in SAP of ≥ 20 mmHg or diastolic arterial pressure (DAP) ≥ 10 mmHg during sitting or standing when compared with supine rest prior to mobilization. Patients were classified as having OI if they experienced dizziness, nausea, blurred vision, feeling of heat, pre-syncope during sitting or standing or syncope, regardless of blood pressure[2, 3] using a standardized questionnaire (Additional file 1: Table S1). Patients not being able to complete the mobilization procedure due to unbearable OI symptoms were classified as having severe OI, regardless of blood pressure.

### Data collection

The finger arterial pressure curve and derived cardiovascular values were analyzed with LiDCOviewPro version 1.1 software (LiDCO, London, UK). NIRS and PI curves were analyzed using MasimoTrace™. Each curve was visually inspected for artefacts before averaging, and such data were excluded. During the supine rest period, values were averaged over 5 min, while periods of PLR, sitting and standing were averaged over the last 10 s before termination of each posture, both in patients completing and terminating the mobilization procedure prematurely.

### Statistical analysis

All data were evaluated for normal distribution by Q-Q plots and histograms before analysis. Normally and non-normally distributed continuous variables are presented as mean, standard deviation (SD) and median, inter-quartile range [IQR], respectively. Categorical variables were reported as frequency with percentages. Differences in patients’ characteristics, peri- and postoperative variables between OT (orthostatic tolerant), OI, and severe OI patients were identified using unpaired t-test or Mann–Whitney U-test. A mixed model analysis of variance (ANOVA) for repeated measures was used for comparison of cardiovascular variables within each test session and between OT, OI and severe OI patients. Statistical analysis was carried out in SPSS version 25 (IBM Corp., Troy, NY, USA). A two-sided P < 0.05 was considered statistically significant.

### Sample size calculation

Previous observations on OI in TKA patients in our department showed an incidence of ~ 40% [8]. We assumed a lower OI incidence in UKA patients due to limited blood loss and assumed minor surgical trauma. To estimate an assumed absolutely reduced incidence to 20% (relative reduction of 50%) with 95%CI of 10–40% compared to major knee surgery, we needed 38 patients. To account for possible dropouts, we wanted to include 42 patients. However, due to COVID 19 restrictions, we could only enroll 38 patients in our study.

## Results

**Fig. 1:**
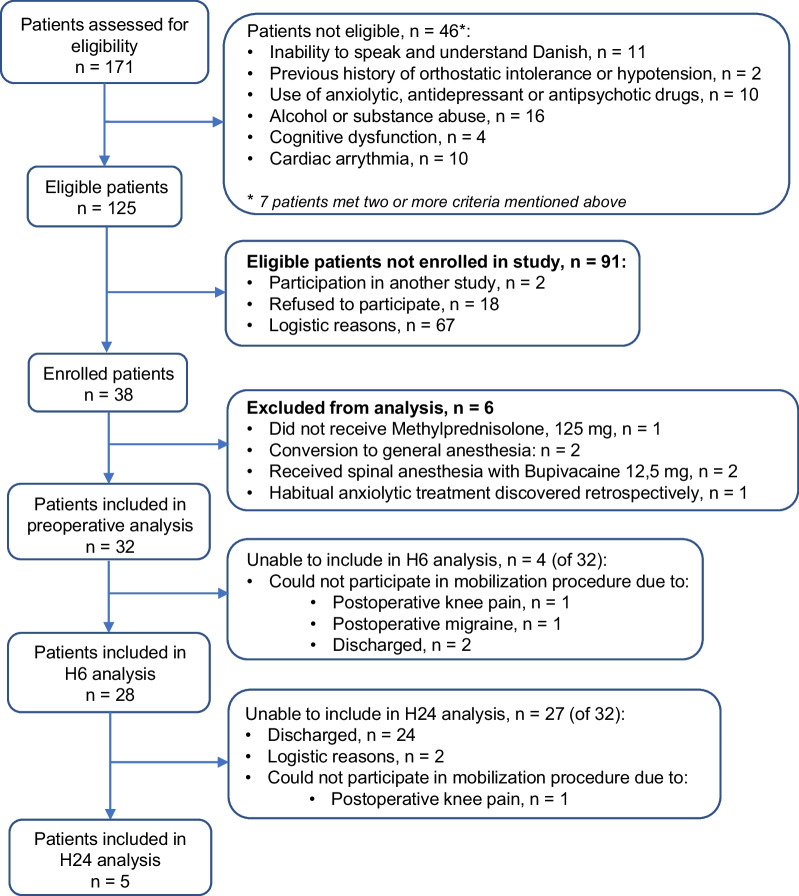
STROBE flow chart

Data on patient flow and exclusion reasons are presented in Consort diagram in Fig. 1. Thirty-eight patients were enrolled in the study, of which 32 patients were included in the preoperative analysis, 28 patients in 6 h postoperative analysis and five patients in 24 h postoperative analysis (Fig. 1).

Pre- and intraoperative characteristics are presented in Table 1.

**Table 1:** Preoperative and intraoperative characteristics

Variables	Mean (SD), median [IQR], No., {%}
Preoperatively (n = 32)	
Age (years)	68 (8)
Weight (kg)	86 (19)
Height (cm)	171 (9)
BMI (kg m2)	29 (5)
Patients receiving antihypertensives	18 {56}
Patients receiving opioids	7 {22}
Patients with DM type II	2 {6}
Patients with cardiovascular disease	21 {66}
Patients with ASA score I-II	30 {94}
Patients with ASA score III	2 {6}
Preoperative hemoglobin (mmol/L)	8.6 (0.6)
Preoperative CRP (mmol/L)	2 (2)
Preoperative pain in supine position (VRS)	0 [0]
Preoperative pain in sitting position (VRS)	0 [0]
Preoperative pain in standing position (VRS)	0 [1]
Intraoperative (n = 32)	
Surgery duration (min)	58 (8)
Bleeding (mL)	0 [25]
Intraoperative fluid balance (mL)	893 (281)
Ringer acetate (mL)	632 (211)
Propofol (mg)	126 [263]
Ephedrine (mg)	0 [10]
Phenylephrine (mg)	0 [0]
Sufentanil (microgram)	0 [10]

Data presented as mean (SD), median [IQR] for continuous measures or number {proportion} for categorical measures

### Orthostatic hypotension and intolerance

Thirty-two patients were mobilized prior to surgery, of these two patients (6%) presented with OH; however, no patients experienced symptoms of OI.

At 6 h postoperatively, two patients were already discharged. Out of 28 patients mobilized at 6 h postoperatively, four (14%, 95%CI 4–33%) patients experienced OI symptoms, and two of these presented with concomitant OH. No patients experienced severe OI. Furthermore, two patients (7%) experienced OH without OI symptoms.

At 24 h after surgery, 24 (75%) patients were already discharged. Of the five patients available for measurements, two patients (40%) experienced OI without OH. One patient presented with severe OI and terminated the mobilization procedure prematurely due to intolerable dizziness. This patient also experienced OI during mobilization at 6 h received b-blocker treatment. No patients experienced isolated OH during mobilization.

Data on frequency and type of OI symptoms are presented in Table 2.

**Table 2:** Number of patients experiencing OI symptoms during mobilization procedure at 6 h and 24 h postoperatively

OI-symptoms	No., (%)	
	6 h, n = 28	24 h, n = 5
Dizziness	2 (7)	1 (20)
Nausea	0 (0)	0 (0)
Vomiting	0 (0)	0 (0)
Blurred vision	0 (0)	0 (0)
Feeling of heat	2 (7)	1 (20)
Pre-syncope	0 (0)	1 (50)
Concurrence of symptoms in OI patients		
Single OI-symptom	3 (11)	2 (40)
Two OI-symptoms	1 (4)	0 (0)
Three OI-symptoms	0 (0)	0 (0)

Data presented as number (proportion)

*OI* orthostatic intolerance

### Cardiovascular responses

Data on absolute cardiovascular variables during mobilization for the entire cohort prior to, at 6 and 24 h after surgery are presented in Additional file 2: Table S2.

### Preoperatively

**Fig. 2:**
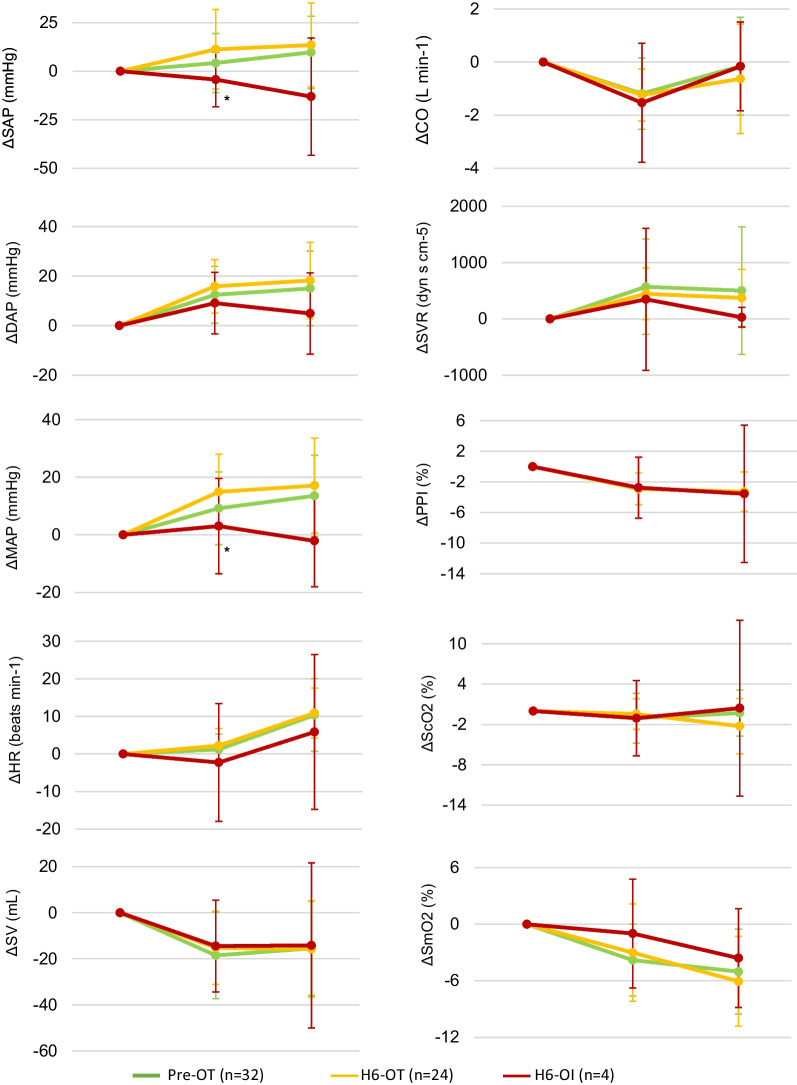
Changes in cardiovascular variables preoperatively (Pre) and 6 h post-surgery (H6) in orthostatic tolerant (OT) and orthostatic intolerant (OI) patients during a standardized mobilization procedure; All data presented as mean (SD) or median (IQR): *p ≤ 0.05 compared with OT patients at 6 h postoperatively

Data on cardiovascular responses during the mobilization procedure prior to surgery are presented in Fig. 2. Six patients (19%) had a rise in SV > 10% during PLR, none of which presented with OI symptoms during mobilization.

### Six hours postoperatively

Data on relative cardiovascular responses during mobilization at 6 h after surgery are presented in Table 3 and Fig. 1. When compared to OT patients, OI patients presented with significantly attenuated cardiovascular responses in sitting position in SAP (11 (20) vs. − 4 [14] mmHg; *p* = 0.02) and MAP (15 (13) vs. 3 [17] mmHg; *p* = 0.04). Furthermore, OI patients presented with attenuated responses in standing position in SAP (13 (22) vs − 13 [] mmHg, *p* = 0.06), DAP (18 (15) vs. 5 [16] mmHg; *p* = 0.10), MAP (17 (16) vs. − 2 [16] mmHg; *p* = 0.05) and SVR (372 (506) vs. 27 [176] dyn s cm^−5^; *p* = 0.10) but these did not reach statistical significance. There were no statistically significant differences in changes in tissue oxygenation and peripheral perfusion index values between OI and OT patients.

**Table 3:** Changes in cardiovascular variables during sitting and standing position grouped by orthostatic tolerance during mobilization procedure

Tolerance status	Pre	6 h after surgery			24 h after surgery		
	OT	OT	OI	*p* value	OT	OI	*p* value
Patients, no. (%)	32 (100)	24 (86)	4 (14)		3 (60)	2 (40)	
Supine to sitting							
∆SAP (mmHg)	4 (15)	11 (20)	− 4 [14]*	0.02	2 [.]	7 [.]	0.40
∆DAP (mmHg)	12 (11)	16 (11)	9 [12]	0.50	14 [.]	11 [.]	1.00
∆MAP (mmHg)	9 (13)	15 (13)	3 [17]*	0.04	8 [.]	11 [.]	1.00
∆HR (beats min^−1^)	1 (4)	2 (5)	− 2 [16]	0.64	1 [.]	0 [.]	1.00
∆SV (mL)	− 19 (19)	− 15 (16)	− 14 [20]	1.00	− 30 [.]	− 6 [.]	0.40
∆CO (litre min^−1^)	− 1.2 (1.3)	− 1.2 (0.9)	− 1.5 [2.2]	0.78	− 1.6 [.]	− 0.5 [.]	0.40
∆SVR (dyn s cm^−5^)	570 (844)	445 (458)	347 [1261]	0.83	347 [.]	556 [.]	1.00
∆PPI (%)	− 2 (2)	− 3 (2)	− 3 [4]	0.97	− 3 [.]	− 3 [.]	0.40
∆ScO2 (%)	− 1 (4)	0 (2)	− 1 [6]	0.66	1 [.]	− 1 [.]	0.20
∆SmO2 (%)	− 4 (4)	− 3 (5)	− 1 [6]	0.70	− 1 [.]	− 1 [.]	1.00
Supine to standing							
∆SAP (mmHg)	10 (19)	13 (22)	− 13 [30]	0.06	4 [.]	22 [.]	0.80
∆DAP (mmHg)	15 (15)	18 (15)	5 [16]	0.10	10 [.]	14 [.]	1.00
∆MAP (mmHg)	14 (14)	17 (16)	− 2 [16]	0.05	9 [.]	23 [.]	0.40
∆HR (beats min^−1^)	10 (10)	11 (7)	6 [21]	0.51	9 [.]	14 [.]	0.20
∆SV (mL)	− 15 (20)	− 16 (21)	− 14 [36]	0.98	− 11 [.]	2 [.]	0.80
∆CO (litre min^−1^)	− 0.2 (1.8)	− 0.6 (2.1)	− 0.2 [1.7]	0.64	− 0.1 [.]	1.6 [.]	0.80
∆SVR (dyn s cm^−5^)	501 (1132)	372 (506)	27 [176]	0.10	86 [.]	26 [.]	0.80
∆PPI (%)	− 2 (2)	− 3 (3)	− 4 [9]	0.92	− 3 [.]	− 6 [.]	0.20
∆ScO2 (%)	0 (3)	− 2 (4)	0 [13]	0.52	0 [.]	0 [.]	0.80
∆SmO2 (%)	− 5 (5)	− 6 (5)	− 4 [5]	0.64	− 2 [.]	− 3 [.]	0.67

Data presented as mean (SD) or median [IQR]

*SAP* systolic arterial pressure, *DAP* diastolic arterial pressure, *MAP* mean arterial pressure, *HR* heart rate, *SV* stroke volume, *CO* cardiac output, *SVR* systemic vascular resistance, *PP* pulse pressure, *PPI* peripheral perfusion index, *ScO*_*2*_ cerebral tissue oxygenation, *SmO*_*2*_ muscular tissue oxygenation, *OT* orthostatic tolerant patients, *OI* orthostatic intolerant patients

* p < 0.05 was statistically significant

No patients were classified as preload dependent.

### Twenty-four hours postoperatively

Changes in cardiovascular variables during mobilization at 24 h after surgery are presented in Table 3. There were no statistically significant changes in cardiovascular variables during mobilization between OT and OI patients (*p* > 0.20).

Two patients (40%) were classified as preload dependent, one of which experienced OI.

### Patient characteristics, pre-, intra- and postoperative variables

There were no statistically significant differences between OT and OI patients in gender, age, BMI, usage of habitual antihypertensives or opioids and ASA score.

Furthermore, differences in pre-, intra- and postoperative variables between OT and OI patients at 6 and 24 h after surgery are presented in Table 4. There were no statistically significant differences between OT and OI patients.

**Table 4:** Pre-, intra- and postoperative variables grouped by orthostatic tolerance during the mobilization procedure

Tolerance status	6 h after surgery			24 h after surgery		
	OT	OI	*p* value	OT	OI	*p* value
Patients, no. (%)	24 (86)	4 (14)		3 (60)	2 (40)	
Preoperative values						
Preoperative hemoglobin (mmol/L)	8.6 (0.6)	9.1 [1.2]	0.32	8.8 [.]	8.2 [.]	0.20
Preoperative CRP (mmol/L)	2 (2)	2 [2]	0.50	3 [.]	1 [.]	0.20
Intraoperative values						
Bleeding during surgery (mL)	0 [24]	0 [113]	0.98	0 [.]	0 [.]	0.80
Surgery duration (min)	58 (8)	57 [18]	0.98	70 [.]	50 [.]	0.20
Propofol (mg)	153 (176)	0 [109]	0.21	356 [.]	86 [.]	0.40
Phenylephrine (mg)	0 [0,2]	0 [0]	0.47	0 [0]	0 [0]	1.00
Ephedrine (mg)	0 [10]	15 [16]	0.04	0 [.]	8 [.]	1.00
Postoperative variables						
Bromage score (0–3)	0 (0)	0 [0]	1.00	0 (0)	0 [0]	1.00
Pain in supine position (VRS 0–10)	2 [4]	3 [4]	0.87	1 [.]	4 [.]	0.80
Pain in sitting position (VRS 0–10)	2 [4]	3 [2]	0.57	2 [.]	3 [.]	0.80
Pain in standing position (VRS 0–10)	2 [3]	4 [3]	0.29	5 [.]	4 [.]	0.80
Opioid equivalents 6 h prior to mobilization (mg)	0 [3]	2 [7]	0.47	0 [.]	3 [.]	0.80
Cumulated opioid equivalents prior to mobilization (mg)	0 [3]	2 [7]	0.47	15 [.]	3 [.]	0.40
Chlorzoxazone 4 h prior to mobilization (mg)	0 [0]	0 [186]	0.73	0 [.]	125 [.]	0.80
Hemoglobin 6 h after surgery (mmol/L)	8.3 (0.8)	9.1 [1.5]	0.25			
CRP 6 h after surgery (mmol/L)	2 (1)	3 [2]	0.82			
Hemoglobin 24 h after surgery (mmol/L)				8.0 [.]	7.6 [.]	0.20
CRP 24 h after surgery (mmol/L)				46 [.]	16 [.]	0.20

Data presented as mean (SD) or median [IQR]

*OT* orthostatic tolerant patients, *OI* orthostatic intolerant patients

Logistic regression analysis showed that pain in a standing position (*p* = 0.41) and opioid usage prior to mobilization (*p* = 0.49) were not associated with OI at 6 h postoperatively.

## Discussion

This single-center prospective observational cohort study’s key finding is the low incidence of early OI after UKA of ~ 15%, compared to previously described incidence of ~ 40% after the more extensive TKA procedure [8]. No patients experienced severe OI preventing mobilization, hence all patients completed the mobilization procedure. One patient experienced severe OI at 24 h postoperatively. OI was associated with attenuated orthostatic responses in SAP and MAP even in sitting position, as shown in other procedures [7, 9].

Unicompartmental knee arthroplasty (UKA) is a commonly performed orthopedic procedure with generally accepted indications and increasing implementation [12]. UKA is especially interesting in the OI context as it is perceived to be a less extensive surgical procedure compared to TKA. This is supported by observations of minimal intraoperative blood loss, lower postoperative pain and opioid usage, less morbidity and faster postoperative recovery [10, 12, 14, 16]. Consequently, UKA is followed by a higher same-day discharge ratio compared to TKA [13].

The stress response to surgery entails neuroendocrine-metabolic and inflammatory-immune mechanisms. Accordingly, it depends on the extent, invasiveness, and duration of the surgical procedure [23]. Even though the magnitude of the surgical stress response after UKA is not examined per se, less tissue is resected, and more anatomical structures are preserved. The low OI incidence we report in the current study is in line with the scarce number of studies exploring OI after minor surgical procedures. A single prospective observational study reported an OI incidence of 4% after superficial breast cancer surgery [9], while a recent retrospective study examining OI incidence and risk factors after knee arthroplasty only included eight UKA patients and reported OI in a single patient [17]. In contrast, numerous studies have described OI incidence of 40–60% after major surgical procedures, such as total knee arthroplasty [8], total hip arthroplasty [7, 24, 25], laparoscopic colorectal resection [5], radical prostatectomy [6, 26], gastrectomies [27], laparoscopic gynecologic surgery [28] and various cardiothoracic procedures [28, 29]. Hence, the findings in the current study further contribute to the notion that the severity of the surgical stress response plays a significant role in the development of OI.

Opioids may increase parasympathetic and decrease sympathetic outflow, potentially resulting in depression of cardiovascular responses [30, 31]. Hence, postoperative pain treatment with opioids may be a relevant contributing factor to postoperative OI, independent of inflammation, pain, and blood loss [32]. UKA is associated with significantly lower postoperative pain scores and opioid consumption compared to TKA [14]. Accordingly, we found low postoperative pain scores during mobilization and minimal opioid postoperative usage. Furthermore, there were no significant differences in these variables between OT and OI patients.

Mild acute blood loss might contribute to OI [33], independently of postoperative inflammation, opioid usage and pain. Unsurprisingly, tourniquet-assisted UKA is associated with lower visible blood loss and smaller hemoglobin drop compared to TKA [34]. Hence, we observed negligible blood loss and minor hemoglobin drop in our patients and no significant differences between OT and OI patients. We assessed preload dependency by PLR prior to mobilization, but there was no association between SV > 10% and OI symptoms onset during mobilization.

A recent study investigating OI after TKA reported a high OI incidence of 44% and 22% at 6 and 24 h, respectively, whilst only 12% of patients were discharged at 24 h [8]. In addition to the lower incidence found in the current UKA study, our data also show that 75% of UKA patients were already discharged 24 h after surgery. Under the assumption that all discharged patients were orthostatic tolerant according to discharge criteria, the presumed OI incidence at 24 h in our cohort would only be 7%. These findings are in line with a recent study without OI/OH data reporting that UKA patients, compared with matched TKA patients, had a shorter median hospital length of stay and a higher rate of discharge on the day of surgery [13]. Increased in-hospital length of stay due to OI has previously been described in other types of major surgery, such as laparoscopic colorectal resection [5], radical prostatectomy [26] and total hip arthroplasty [35].

We observed significantly impaired responses in SAP and MAP in sitting positions in orthostatic intolerant patients. Contrasting previous studies [6–8], the attenuated responses in SAP, DAP, MAP, HR and SVR in standing position did not reach statistical significance, probably due to small sample size of patients experiencing OI. We also did not observe significant differences in ScO2 and PPI responses between orthostatic tolerant and intolerant patients. These findings further support the notion that OI is the final common pathway for diverse pathophysiological pathways in heterogeneous populations. Finally, OI is not always accompanied by cardiovascular perturbances such as OH, as previously described [7, 8].

The Bromage test [22] was performed to evaluate residual motor blockade before mobilization, as all patients received spinal anaesthesia with 10 mg hyperbaric Bupivacaine. Furthermore, all patients were able to be mobilized at 6 h postoperatively. Nevertheless, a residual vasomotor block cannot be ruled out by the Bromage test. However, a high incidence of postoperative OI is previously described in patients undergoing general anaesthesia [5, 6], suggesting a different pathophysiological pathway.

Finally, although UKA and TKA share a common indication, patient selection bias might occur when deciding on the surgical procedure[36]. Even if our findings are confounded by selection bias, they would only further support the notion of multifactorial etiology of postoperative orthostatic intolerance, as patient-related factors likely also contribute to its development.

This is, to the best of our knowledge, the first study to report in detail hemodynamic and tissue oxygenation changes during early mobilization in fast-track patients undergoing UKA. Further strengths comprise standardized perioperative care protocols, including surgery and analgesia mobilization procedure and symptom questionnaires. There are several limitations to our study. The biggest limitation is the small sample size, further challenged by the COVID19-restriction, resulting in imprecision of the incidence estimate. Furthermore, our study was not intended to explore associations of secondary outcomes nor demonstrate causality.

In conclusion, we describe a low incidence of early postoperative OI following fast-track UKA, associated to decreased orthostatic pressure responses.

### Supplementary Information

**Additional file 1: Table S1**. Absolute cardiovascular variables during mobilization procedure before, at 6h and 24h after surgery.

**Additional file 2: Table S2.** Standardized questionnaire for symptoms of orthostatic intolerance.

## Data Availability

Original data and datasets generated and/or analyzed during the current study are stored on a Region Hovedstadens secured research drive but are not publicly available due to individual privacy protection. Data are available from the corresponding author on reasonable request.

## Abbreviations

ASA Score: American Society of Anesthesiologists
CO: Cardiac output
DAP: Diastolic arterial pressure
ERAS: Early recovery after surgery
HR: Heart rate
MAP: Mean arterial pressure
NIRS: Near-InfraRed spectroscopy
OI: Orthostatic intolerance
OH: Orthostatic hypotension
PACU: Post-anesthesia care unit
PI: Perfusion Index
PLR: Passive leg raise
PO: Per Os
SAP: Systolic arterial pressure
ScO_2_: Cerebral oxygenation
SmO_2_: Muscular oxygenation
SV: Stroke volume
SVR: Systemic vascular resistance
TKA: Total knee arthroplasty
UKA: Unicompartmental knee arthroplasty

## References

[1] Kehlet H (1997). Multimodal approach to control postoperative pathophysiology and rehabilitation. Br J Anaesth.

[2] Consensus statement on the definition of orthostatic hypotension, pure autonomic failure, and multiple system atrophy. The Consensus Committee of the American Autonomic Society and the American Academy of Neurology. Neurology. 1996;46(5):1470. 10.1212/wnl.46.5.1470 8628505

[3] Freeman R, Wieling W, Axelrod FB, Benditt DG, Benarroch E, Biaggioni I (2011). Consensus statement on the definition of orthostatic hypotension, neurally mediated syncope and the postural tachycardia syndrome. Clin Auton Res.

[4] Jans Ø, Kehlet H (2017). Postoperative orthostatic intolerance: a common perioperative problem with few available solutions. Can J Anesth.

[5] Eriksen JR, Munk-Madsen P, Kehlet H, Gögenur I (2019). Orthostatic intolerance in enhanced recovery laparoscopic colorectal resection. Acta Anaesthesiol Scand.

[6] Bundgaard-Nielsen M, Jørgensen CC, Jørgensen TB, Ruhnau B, Secher NH, Kehlet HF (2009). Orthostatic intolerance and the cardiovascular response to early postoperative mobilization. Br J Anaesth.

[7] Jans Ø, Bundgaard-Nielsen M, Solgaard S, Johansson PIKH (2012). Orthostatic intolerance during early mobilization after fast-track hip arthroplasty. Br J Anaesth.

[8] Hristovska A-M, Andersen LB, Grentoft M, Mehlsen J, Gromov K, Kehlet H (2022). Orthostatic intolerance after fast-track knee arthroplasty: Incidence and hemodynamic pathophysiology. Acta Anaesthesiol Scand.

[9] Müller RG, Bundgaard-Nielsen M, Kehlet H (2010). Orthostatic function and the cardiovascular response to early mobilization after breast cancer surgery. Br J Anaesth.

[10] Price AJ, Alvand A, Troelsen A, Katz JN, Hooper G, Gray A (2018). Knee replacement. Lancet.

[11] Mikkelsen M, Price A, Pedersen AB, Gromov K, Troelsen A (2022). Optimized medial unicompartmental knee arthroplasty outcome: learning from 20 years of propensity score matched registry data. Acta Orthop.

[12] Crawford DA, Berend KR, Thienpont E (2020). Unicompartmental knee arthroplasty: US and global perspectives. Orthop Clin N Am.

[13] Jensen CB, Petersen PB, Jørgensen CC, Kehlet H, Troelsen A, Gromov K (2021). Length of stay and 90-day readmission/complication rates in unicompartmental versus total knee arthroplasty: a propensity-score-matched study of 10,494 procedures performed in a fast-track setup. J Bone Joint Surg Am.

[14] Leiss F, Götz JS, Maderbacher G, Zeman F, Meissner W, Grifka J (2020). Pain management of unicompartmental (UKA) vs total knee arthroplasty (TKA) based on a matched pair analysis of 4144 cases. Sci Rep.

[15] Beard DJ, Davies LJ, Cook JA, MacLennan G, Price A, Kent S (2019). The clinical and cost-effectiveness of total versus partial knee replacement in patients with medial compartment osteoarthritis (TOPKAT): 5-year outcomes of a randomised controlled trial. Lancet.

[16] Liddle AD, Judge A, Pandit H, Murray DW (2014). Adverse outcomes after total and unicompartmental knee replacement in 101,330 matched patients: a study of data from the National Joint Registry for England and Wales. Lancet.

[17] Kurkis GM, Dennis DA, Johnson RM, Mejia M, Yazdani-Farsad Y, Jennings JM (2022). Incidence and risk factors of orthostasis after primary hip and knee arthroplasty. J Arthroplasty.

[18] Gromov K, Grassin-Delyle S, Foss NB, Pedersen LM, Nielsen CS, Lamy E (2021). Population pharmacokinetics of ropivacaine used for local infiltration anaesthesia during primary total unilateral and simultaneous bilateral knee arthroplasty. Br J Anaesth.

[19] Maizel J, Airapetian N, Lorne E, Tribouilloy C, Massy Z, Slama M (2007). Diagnosis of central hypovolemia by using passive leg raising. Intensive Care Med.

[20] de Wilde RBP, Geerts BF, van den Berg PCM, Jansen JRC (2009). A comparison of stroke volume variation measured by the LiDCOplus and FloTrac-Vigileo system. Anaesthesia.

[21] Monnet X, Teboul J-L (2015). Passive leg raising: five rules, not a drop of fluid!. Crit Care.

[22] McNamee DA, McClelland AM, Scott S, Milligan KR, Westman L, Gustafsson U (2002). Spinal anaesthesia: comparison of plain ropivacaine 5 mg ml(-1) with bupivacaine 5 mg ml(-1) for major orthopaedic surgery. Br J Anaesth.

[23] Cusack B, Buggy DJ (2020). Anaesthesia, analgesia, and the surgical stress response. BJA Educ.

[24] Jans O, Mehlsen J, Kjærsgaard-Andersen P, Husted H, Solgaard S, Josiassen J (2015). Oral midodrine hydrochloride for prevention of orthostatic hypotension during early mobilization after hip arthroplasty: a randomized, double-blind, placebo-controlled trial. Anesthesiology.

[25] Lindberg-Larsen V, Petersen PB, Jans Q, Beck T, Kehlet H (2018). Effect of pre-operative methylprednisolone on orthostatic hypotension during early mobilization after total hip arthroplasty. Acta Anaesthesiol Scand.

[26] Bundgaard-Nielsen M, Jans Ø, Müller RG, Korshin A, Ruhnau B, Bie P (2013). Does goal-directed fluid therapy affect postoperative orthostatic intolerance? A randomized trial. Anesthesiology.

[27] Park KO, Lee YY (2013). Orthostatic intolerance ambulation in patients using patient controlled analgesia. Korean J Pain.

[28] Mizota T, Iwata Y, Daijo H, Koyama T, Tanaka T, Fukuda K (2013). Orthostatic intolerance during early mobilization following video-assisted thoracic surgery. J Anesth.

[29] Hanada M, Tawara Y, Miyazaki T, Sato S, Morimoto Y, Oikawa M (2017). Incidence of orthostatic hypotension and cardiovascular response to postoperative early mobilization in patients undergoing cardiothoracic and abdominal surgery. BMC Surg.

[30] Hristovska AM, Uldall‐Hansen B, Mehlsen J, Kehlet H, Foss NB (2023). Orthostatic intolerance after intravenous administration of morphine: incidence haemodynamics and heart rate variability analysis. Anaesthesia.

[31] Headrick JP, Pepe S, Peart JN (2012). Non-analgesic effects of opioids: cardiovascular effects of opioids and their receptor systems. Curr Pharm Des.

[32] Hristovska AM, Uldall-Hansen B, Mehlsen J, Kehlet H, Foss NB (2023). Orthostatic intolerance after intravenous administration of morphine: incidence, haemodynamics and heart rate variability analysis. Anaesthesia.

[33] Hristovska A-M, Uldall-Hansen B, Mehlsen J, Andersen LB, Kehlet H, Foss NB (2023). Orthostatic intolerance after acute mild hypovolemia: incidence, pathophysiologic hemodynamics, and heart-rate variability analysis-a prospective observational cohort study. Can J Anaesth.

[34] Schwab P-E, Lavand’homme P, Yombi JC, Thienpont E (2015). Lower blood loss after unicompartmental than total knee arthroplasty. Knee Surg Sports Traumatol Arthrosc.

[35] Skarin MU, Rice DA, McNair PJ, Kluger MT (2019). Orthostatic intolerance following hip arthroplasty: incidence, risk factors and effect on length of stay: a prospective cohort study. Eur J Anaesthesiol.

[36] Howell RE, Lombardi AVJ, Crilly R, Opolot S, Berend KR (2015). Unicompartmental knee arthroplasty: Does a selection bias exist?. J Arthroplasty.

