# Supplementary material for: Postoperative orthostatic intolerance following fast-track unicompartmental knee arthroplasty: incidence and hemodynamics—a prospective observational cohort study
Source: J Orthop Surg Res. 2024 Apr 1;19:214. doi: 10.1186/s13018-024-04639-6 (PMC10983746; doi:10.1186/s13018-024-04639-6)
Supplement: Supplementary file 1 — Additional file 1: Table S1. Absolute cardiovascular variables during mobilization procedure before, at 6h and 24h after surgery. [file 13018_2024_4639_MOESM1_ESM.docx]

Additional table 1 – Standardized questionnaire for symptoms of orthostatic intolerance

| **^Symptoms of orthostatic intolerance^** | ^Pre^  ^Supine^ | ^Sit^ | ^Stand^ | ^H6^  ^Supine^ | ^Sit^ | ^Stand^ | ^H24^  ^Supine^ | ^Sit^ | ^Stand^ |
| --- | --- | --- | --- | --- | --- | --- | --- | --- | --- |
| ^Dizziness^ |  |  |  |  |  |  |  |  |  |
| ^Nausea^ |  |  |  |  |  |  |  |  |  |
| ^Visual disturbances^ |  |  |  |  |  |  |  |  |  |
| ^Feeling of heat^ |  |  |  |  |  |  |  |  |  |
| ^Vomiting^ |  |  |  |  |  |  |  |  |  |
| ^Pre-syncope^ |  |  |  |  |  |  |  |  |  |
| ^Syncope^ |  |  |  |  |  |  |  |  |  |
|  | | | | | | | | | |
| ***^Comments^*** |  |  |  |  |  |  |  |  |  |
| ***^Investigator^*** |  |  |  |  |  |  |  |  |  |
| ***^Date & time^*** |  |  |  |  |  |  |  |  |  |

*^Green = Tolerable symptoms^*

***^Red = Intolerable symptoms^***
