# Supplementary material for: Postoperative orthostatic intolerance following fast-track unicompartmental knee arthroplasty: incidence and hemodynamics—a prospective observational cohort study
Source: J Orthop Surg Res. 2024 Apr 1;19:214. doi: 10.1186/s13018-024-04639-6 (PMC10983746; doi:10.1186/s13018-024-04639-6)
Supplement: Supplementary file 2 — Additional file 2: Table S2. Standardized questionnaire for symptoms of orthostatic intolerance. [file 13018_2024_4639_MOESM2_ESM.docx]

Additional table 2 – Absolute cardiovascular variables during mobilization procedure before, at 6 and 24h after surgery ^1^

|  | **^Preoperatively, n =32^** | | | | | **^6h postoperatively , n=28^** | | | | | **^24h postoperatively, n = 5^** | | | | |
| --- | --- | --- | --- | --- | --- | --- | --- | --- | --- | --- | --- | --- | --- | --- | --- |
|  | **^Supine 1^** | **^PLR^** | **^Supine 2^** | **^Sit^** | **^Stand^** | **^Supine 1^** | **^PLR^** | **^Supine 2^** | **^Sit^** | **^Stand^** | **^Supine 1^** | **^PLR^** | **^Supine 2^** | **^Sit^** | **^Stand^** |
| **^SAP (mmHg)^** | ^141 (15)^ | ^141 (17)^ | ^138 (22)^ | ^142 (30)^ | ^148 (32) #^ | ^145 (21)^ | ^141 (21) *^ | ^140 (21)^ | ^149 (24)^ | ^152 (31)^ | ^142 [18]^ | ^149 [13]^ | ^145 [21]^ | ^150 [27]^ | ^152 [23]^ |
| **^DAP (mmHg)^** | ^73 (12)^ | ^71 (13)^ | ^70 (14)^ | ^82 (21) #^ | ^85 (22) #^ | ^72 (14)^ | ^70 (15)^ | ^70 (15)^ | ^85 (19) #^ | ^87 (20) #^ | ^66 [28]^ | ^68 [26]^ | ^69 [25]^ | ^83 [37] #^ | ^79 [35] #^ |
| **^MAP (mmHg)^** | ^101 (12)^ | ^101 (13)^ | ^99 (15)^ | ^108 (23) #^ | ^113 (25) # $^ | ^101 (16)^ | ^99 (16) *^ | ^99 (16)^ | ^112 (20) #^ | ^114 (22) #^ | ^98 [29]^ | ^97 [29]^ | ^97 [25]^ | ^104 [34] #^ | ^110 [36] #^ |
| **^HR (beats min-1)^** | ^68 (13)^ | ^68 (12)^ | ^67 (12)^ | ^68 (12)^ | ^77 (14) # $^ | ^86 (16)^ | ^86 (15)^ | ^85 (17)^ | ^88 (15)^ | ^97 (17) # $^ | ^69 [25]^ | ^70 [33]^ | ^68 [31]^ | ^69 [34]^ | ^78 [22] $^ |
| **^SV (mL)^** | ^90 (31)^ | ^93 (32) *^ | ^92 (35)^ | ^74 (27) #^ | ^77 (37) #^ | ^89 (28)^ | ^89 (30)^ | ^86 (31)^ | ^71 (25) #^ | ^70 (27) #^ | ^114 [70]^ | ^127 [63]^ | ^119 [61]^ | ^89 [64]^ | ^106 [74]^ |
| **^CO (litre min-1)^** | ^6,1 (2,5)^ | ^6,3 (2,4) *^ | ^6,2 (6,0)^ | ^5,0 (2,1) #^ | ^6,1 (3,3) $^ | ^7,6 (3,2)^ | ^7,8 (3,3)^ | ^7,4 (3,1)^ | ^6,1 (2,3) #^ | ^6,8 (3,0)^ | ^7, [4,6]^ | ^6,8 [5,4]^ | ^7,7 [3,7]^ | ^6,6 [2,3]^ | ^8,1 [4,1] $^ |
| **^SVR (Dyn s cm-1)^** | ^1470 [721]^ | ^1308 [874] *^ | ^1333 [909]^ | ^1751 [1323] #^ | ^1752 [1324] #^ | ^1087 [504]^ | ^1054 [584]^ | ^1091 [556]^ | ^1525 [913] #^ | ^1349 [1046] #^ | ^845 [797]^ | ^981 [802]^ | ^826 [613]^ | ^1189 [1191] #^ | ^869 [888] $^ |
| **^PP (mmHg)^** | ^69 (13)^ | ^70 (14)^ | ^68 (18)^ | ^60 (20) #^ | ^63 (20)^ | ^72 (21)^ | ^71 (18)^ | ^70 (19)^ | ^64 (18)^ | ^65 (22)^ | ^78 [24]^ | ^79 [25]^ | ^80 [39]^ | ^60 [47]^ | ^75 [25]^ |
| **^PPI^** | ^6 (4)^ | ^6 (4)^ | ^6 (4)^ | ^4 (4) #^ | ^4 (4) #^ | ^9 (4)^ | ^8 (4)^ | ^9 (4)^ | ^6 (3) #^ | ^5 (3) #^ | ^10 [1]^ | ^9 [4]^ | ^9 [3]^ | ^5 [2]^ | ^5 [2]^ |
| **^ScO2 (%)^** | ^73 (6)^ | ^73 (8)^ | ^72 (8)^ | ^71 (7)^ | ^70 (7) # $^ | ^74 (6)^ | ^74 (6)^ | ^74 (6)^ | ^74 (6)^ | ^72 (7)^ | ^72 [15]^ | ^72 [15]^ | ^69 [15]^ | ^69 [16]^ | ^69 [16]^ |
| **^SmO2 (%)^** | ^77 (11)^ | ^78 (10) *^ | ^77 (10)^ | ^73 (13) #^ | ^72 (13) #^ | ^79 (5)^ | ^81 (4) *^ | ^80 (5)^ | ^77 (7) #^ | ^74 (8) # $^ | ^84 [16]^ | ^82 [16]^ | ^84 [16]^ | ^82 [16]^ | ^75 [18] # $^ |
